# Polylactic Acid/Saqqez Gum Blends for Chewing Gum Applications: Impact of Plasticizers on Thermo-Mechanical and Morphological Properties

**DOI:** 10.3390/polym16111469

**Published:** 2024-05-22

**Authors:** Mona Kaveh, Samira Yeganehzad, Mohammad Ali Hesarinejad, Maryam Kiumarsi, Mohammad Reza Abdollahi Moghaddam

**Affiliations:** 1 Research Institute of Food Science and Technology (RIFST), Mashhad 91895-157.356, Iran; kavehm@live.com (M.K.); m.abdollahi@rifst.ac.ir (M.R.A.M.); 2 Department of Nutritional Sciences, Faculty of Life Sciences, University of Vienna, Althanstraβe 14, A-1090 Vienna, Austria; kiumarsimaryam@gmail.com

**Keywords:** blend composite, plasticizer, thermo-mechanical properties, chewing gum

## Abstract

This study investigated a blend of poly (lactic acid) (PLA) and Saqqez gum, with a weight ratio of 70:30, respectively, along with two plasticizers, acetyl tributyl citrate (ATBC) and polyethylene glycol (PEG), at three different concentrations (14%, 16% and 18% by weight of the PLA). The blend was analyzed using differential scanning calorimetry (DSC), scanning electron microscopy (SEM), tensile tests, water-absorption behavior (coefficients of water absorption, sorption, diffusion and permeability of the samples during 240 h) and chemical resistance (exposure to 1 mol/L HCl and 1 mol/L NaOH for 240 h). The desired elastomer blend was then used to prepare natural chewing gum, which was subsequently subjected to texture profile analyzer (TPA) tests and sensory evaluation. The results showed that the addition of both plasticizers increased the tensile properties of the blend. Compared to neat PLA, all the blends exhibited an increase in elongation at break and a decrease in yield strength, with the maximum elongation at break (130.6%) and the minimum yield strength (12.2 MPa) observed in the blend containing 16% ATBC. Additionally, all the thermal attributes studied, including T_g_, T_c_ and T_m_, were lower than those of neat PLA, and the T_g_ values deviated from the values predicted via Fox’s equation. SEM images of the blends confirmed that plasticization improved the homogeneity and distribution of the components in the blend structure. PEG 18% and ATBC 16% exhibit the highest and lowest water-absorption behavior, respectively. Regarding chemical resistance, all blends showed weight gain when exposed to HCl, while no weight loss was observed for resistance to NaOH. The chewing gum sample obtained similar values for the mentioned tests compared to the commercial control sample. Overall, the results indicate that plasticization enhances the structure and performance of the PLA/Saqqez gum blend and further investigation is warranted.

## 1. Introduction

In order to address negative ecological impacts such as dwindling petroleum resources, limited landfill space and concerns over toxic gas emissions during incineration, researchers have been studying biodegradable polymers based on renewable and nontoxic resources [1,2]. Biobased polymers made from biological compounds like nonfossil substances have been found in nature and have potential applications in various industries [3,4,5]. Despite bioplastics accounting for less than 1% of all plastics produced in 2020, which is equivalent to only 1.2 million tonnes, the biopolymers market is expected to grow by 11.28% by the end of 2025 [6].

PLA is a desirable biopolymer owing to its ease of processing, suitable thermal stability and comparable price in comparison to other biopolymers. However, its limited mechanical and barrier properties along with its high brittleness restrict its potential applications [6,7,8]. To overcome these drawbacks, researchers have explored the blending of PLA with other polymers, such as starches [9], gum Arabic [10] and plasticizers, as an efficient and cost-effective modification approach [11]. Surya and colleagues (2020) found that to modify the flexibility of PLA, an effective approach is to incorporate plasticizers, flexible polymers and certain gums or resins as complementary components in a blending process [1].

Blending biopolymers with other polymers is a highly effective way of modification to achieve the desired properties, which significantly enhances their characteristics. The primary objective of developing a new polymer blend comprising two or more components is to achieve the highest performance of the blend without extensively altering the characteristics of its constituents [2]. Blending is a challenging process due to immiscibility between the components, which critically hinders the achievement of improved toughness [12,13], and in some cases, this issue has been overcome by adding compatibilizing agents such as Joncryl [6].

Polysaccharide gums, as natural biopolymers, are abundantly available in nature, and have tremendous industrial applications in various fields [14]. One such natural biopolymer that has been less explored as a blending partner is Saqqez gum, a nontoxic, renewable and biodegradable oleo-gum resin which generally includes polysaccharides, α-pinene, terpenoids and flavonoids [14,15,16] derived from Bene (*Pistacia atlantica* subsp. mutica) trees belonging to the Anacardiaceae family in Iran (the Zagros zone) [17].

Plasticizers, as nonvolatile and low molecular weight compounds often added to a hot-melt extrusion formulation, can also be used to improve the physical and mechanical characteristics of PLA blends by increasing flexibility and decreasing melt viscosity and glass transition temperature (T_g_) without altering the fundamental chemical character of the final blending polymer [18]. Plasticizers have the ability to enhance the mobility and rotation of the polymer chain segments, resulting in increased chain movement. This leads to a decrease in the polymer’s glass transition temperature and melt viscosity, and an increase in its flexibility [19]. The most commonly used method to evaluate the effectiveness of plasticizers is by comparing the glass transition temperature and mechanical properties of the polymer with and without plasticizers, using various types and amounts of plasticizers [20]. Acetyl tributyl citrate (ATBC) and polyethylene glycol (PEG) are commonly used plasticizers for PLA blends and have been investigated in previous studies [21,22,23,24,25,26,27,28,29].

The concept of combining various polymers in specific proportions to create a unique material is fascinating. While not a new idea, researchers have employed diverse strategies to improve the properties of the resulting material. A growing demand for innovative, environmentally friendly and cost-effective polymer blends has emerged due to recent economic challenges. Currently, research is predominantly centered on biopolymers as a promising avenue for producing biodegradable plastics. This is because biopolymers possess renewable properties and are cost-effective, scalable and compatible with living systems. The focus of this study was to investigate the effects of two selected plasticizers, PEG and ATBC, on the concentration of the PLA/Saqqez gum blend to create a new eco-friendly blend material suitable for the food and packaging industries. The research involved analyzing the blend’s morphological, thermal and mechanical properties through scanning electron microscopy (SEM), differential scanning calorimetry (DSC) and tensile testing. Additionally, the study investigated the blend’s water-absorption behavior and chemical resistance. In conclusion, this research highlights the potential of utilizing natural biopolymers and plasticizers to enhance the properties of PLA blends, offering sustainable alternatives to conventional petroleum-based polymers.

## 2. Materials and Methods

### 2.1. Materials

Commercial PLA pellets were purchased provided by Behzist Danesh Narvan Co., Mashhad, Iran, which were the Ingeo^TM^ Biopolymer, 4043D from NatureWorks (Minneapolis, MN, USA). The Saqqez gum derived from the resin of the Baneh tree (*Pistacia atlantica* subsp. mutica) was obtained from the local market in Kurdistan, Iran, specifically from Saqqez production Co. (Van). The plasticizers used in the study, ATBC and PEG-40, were purchased from Sigma–Aldrich Co. (St. Louis, MI, USA), while and JONCRYL^®^ ADR-4368-C, a polymeric chain extender and reactive compatibilizer, was supplied by Behzist Danesh Narvan Co., Mashhad, Iran, sourced from BASF in Germany.

### 2.2. Chemical Composition of Saqqez

The moisture, total ash and crude fat contents of Saqqez gum were determined using the AOAC method. The total protein content was determined by analyzing the nitrogen content using the semi-micro Kjeldahl method [30]. A conversion factor of 6.6, based on early determinations, was used to calculate the crude protein content [31]. The polysaccharide content was determined using the phenol-sulfuric acid method [32].

### 2.3. Sample Preparation

**Figure 1:**
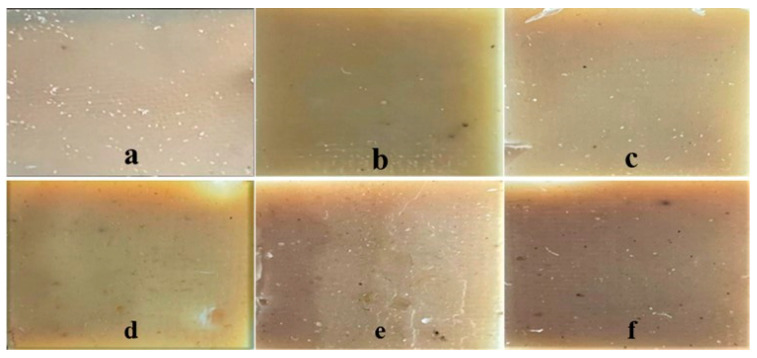
Sample of PPLA/Saqqez gum blends with different types and concentrations of plasticizers (**a**) ATBC 14%, (**b**) ATBC 16%, (**c**) ATBC 18%, (**d**) PEG 14%, (**e**) PEG 16% and (**f**) PEG 18%.

The PLA pellets and Saqqez gum were dried separately overnight in a vacuum oven (Heraeus Vacum therm VT6130P, Borken, Germany) at 80 °C and 45 °C, respectively. To prepare the plasticized PLA (PPLA), ATBC or PEG at different concentrations (14, 16 and 18% *w*/*w* based on PLA) were directly melt-mixed with PLA using an internal mixer (RheoSense, IPPI, Pajoohesh Blvd, Iran) at 160 °C and 60 rpm for 15 min. To create the PPLA/Saqqez gum blends, the prepared PPLA was manually premixed with Saqqez gum at a 70:30 ratio and joncryl (2 phr), and then melt-mixed in the internal mixer at 150 °C and 60 rpm for 10 min. The formulations were then melted between hot plates at 160 °C for 7 min to remove air bubbles and pressed at 160 °C under 20 MPa for 5 min using a laboratory mini hot press (Toyosiki Press, Tokyo, Japan) to obtain sheets with dimensions of 100 × 100 × 0.3 mm. The sheets were conditioned in a desiccator at 25 °C and 53% relative humidity for 7 days. The resulting samples are shown in Figure 1.

### 2.4. Analysis Methods

#### 2.4.1. Mechanical Properties

The tensile tests were conducted in accordance with ASTM D882-02. Each sample was prepared by punching three rectangular strips with dimensions of 50 mm × 13.1 mm × 0.3 mm from preconditioned sheets. Uniaxial tensile testing was performed at room temperature and relative humidity levels of 40% and 60%. Strength at yield, elongation at the break and Young’s modulus were measured using a SPICO twin-column universal testing machine (Model UTM-S500, Tehran, Iran) with a 5-ton load cell. The test was conducted at a crosshead speed of 5 mm/min until tensile failure occurred. The average values of three repetitions of each sample were reported with standard error.

#### 2.4.2. Thermal Properties

The PPLA/Saqqez gum blends underwent thermal analysis using a differential scanning calorimeter (DSC SL800, SPICO, Wuhan, China). The analysis included determining the glass transition temperature (T_g_), melting temperature (T_m_) and crystallization temperature (T_c_). Sample pans were used to hold samples weighing approximately 5 to 15 mg, which were then sealed and introduced into the heating cell of the DSC. They were then heated from 25 to 250 °C at a rate of 5 K/min under a nitrogen atmosphere with a flow rate of 10 mL/min.

In this study, to further assess the T_g_ quantity, an analysis of T_g_ utilizing Fox’s equation (Equation (1)) was also conducted.

(1) $$\frac{1}{T_{g}\;blend}\approx \frac{W_{1}}{T_{g_{1}}}+\frac{W_{2}}{T_{g_{2}}},$$

In this equation, W_1_, W_2_, T_g__1_ and T_g__2_ are the weight of the first compound, the weight of the second compound, the T_g_ of the first compound and the T_g_ of the second compound, respectively [33].

#### 2.4.3. Morphological Properties

The fracture surface’s morphology was analyzed by using field emission scanning electron microscopy (FESEM) at an accelerating voltage of 10 kV. The analysis was conducted on a surface that was vacuum-coated with gold. The FESEM used was a TESCAN model MIRA3 from the Czech Republic.

#### 2.4.4. Water-Absorption Behavior

The study aimed to investigate the water-absorption behaviour of Saqqez gum and PLA/Saqqez blends with and without two plasticizers under distilled water immersion. The ASTM D 570-98 method was used to study the kinetics of water absorption. The specimens, except for Saqqez gum, were dried in an oven at 100 °C for 24 h before measuring their weight. Saqqez gum was dried at 40 °C for the same duration. The specimens were immersed in distilled water at 25 °C and weighed periodically until they reached equilibrium or saturation point, which took between 1 and 10 days. The weight of the sample before and after absorption was measured using an electronic balance with an accuracy of 10^−4^ g. The percentage of water absorption (WA) was calculated using Equation (2).

(2) $$\mathrm{WA}\;\left(\%\right)=\frac{W_{t}-W_{0}}{W_{0}}\times 100,$$

W_0_ and W_t_ indicate the weight (in grams) of the sample before and after being exposed to water for a specific duration (t). The weight is measured on a sample that has been oven-dried [34,35].

The water absorption (%) was calculated at various time intervals and plotted against the square root of immersion time to determine the diffusion coefficient. The diffusion coefficient is calculated by using Equation (3), which involves finding the slope of the curve between water absorption (%) and the square root of immersion time.

(3) $$\mathrm{Diffusion}\;\mathrm{coefficient}\;\left(D\right)=\pi \;(\frac{t^{2}m^{2}}{16W_{\infty }^{2}})$$

The slope of the linear portion of the sorption curve (m) and the initial sample thickness (t) in mm are used to calculate the sorption coefficients related to the equilibrium sorption, as shown in Equation (4).

(4) $$\mathrm{Sorption}\;\mathrm{coefficient}\;\left(S\right)=W_{\infty }/W_{t}$$

The percentage of water uptake at saturation stage and at time t are represented by W_∞_ and W_t_, respectively. The permeability coefficient, which is the net effect of sorption and diffusion coefficient, can be calculated using Equation (5) [35].

(5) $$\mathrm{Permeability}\;\mathrm{coefficient}\;\left(P\right)=D\times S$$

#### 2.4.5. Chemical Resistance

To evaluate the chemical resistance, the dried samples were pre-weighed in a precision electric balance and immersed in 100 mL of 1 mol/L NaOH and 1 mol/L HCl as chemical reagents for varying periods of time (24–240 h). After removing the treated samples, we filtered, dried and weighed them to determine the percentage loss/gain. The percentage chemical resistance (Pcr) was determined by using the weight loss calculation Equation (6) as follows:

(6) $$\left(Pcr\right)=\frac{W_{aci}-T_{i}\;}{T_{i}}\times 100$$

T_i_ represents the initial weight and W_aci_ represents the weight after a certain interval [36].

### 2.5. Preparation of Chewing Gum Sample Containing Desired Elastomer

The chewing gum sample containing 40% by weight of the desired elastomer, 30% by weight of xylitol, 20% by weight of glycerin plasticizer and 10% by weight of calcium carbonate filler was prepared in the internal mixer. All ingredients were prepared and purchased from all types of food grades available on the market. A sample of commercial sugar-free chewing gum containing xylitol was also obtained from the local market.

#### Comparative Tests of Chewing Gum Produced with Commercial Control

- Texture profile analysis (TPA) test

Cylindrical samples with a thickness of 3 cm and a diameter of 1.5 cm were prepared from the produced gum sample and the commercial gum sample, and then the parameters of hardness, cohesiveness, ferrite, gumminess, chew-ability and breaking point were measured using a TA.XT Plus texture analyzer (Stable Micro Systems, Godalming, UK).

- Sensory evaluation

Sensory evaluation of appearance, texture, odor, color and overall acceptability of the samples based on a 5-point hedonic scale with scores from 1 to 5, very bad, bad, moderate, good and very good, were rated by 23 people, including 12 males and 11 females, aged 20 to 55 years, compared to the control sample of commercial chewing gum.

### 2.6. Statistical Analysis

A one-way Analysis of Variance (ANOVA) was used for the statistical analysis. If the differences were considered significant (*p* < 0.05), Duncan’s test was conducted to check for differences between pairs of groups. The statistical analysis was performed using Minitab 16 software.

## 3. Results and Discussion

### 3.1. Chemical Composition of Saqqez

Table 1 presents the percentage chemical composition of Saqqez gum (*Pistacia atlantica* subsp. mutica) in terms of ash, moisture, fat, protein and polysaccharides.

Khoramdareh et al. (2022) reported the moisture, protein, ash and crude fat of Saqqez oleoresin gum to be 18.52 ± 0.02%, 4.49 ± 0.15%, 0.92 ± 0.003% and 0.86 ± 0.01%, respectively [37]. Our results are more or less in agreement with this study. Mirahmadi et al. (2019) also studied *Pistacia atlantica* subsp. Kurdica gum and found the moisture, total ash and protein content to be in the range of 16.03% to 19.26%, 0.86% to 0.94% and 3.97% to 5.05%, respectively [38]. The protein fraction is responsible for the emulsification properties of the gum [39]. Thus, the higher total protein value in the samples of this study indicates a higher emulsifying power compared to previous studies. Mohammadi et al. (2019) reported the ash contents of various gums as follows: flaxseed (7.4%), xanthan (9.35%), guar (11.9%), locust bean (0.7%), karaya (3.4%) and Arabic (1.2%) [16,38]. Therefore, it is possible to compare them and see that Saqqez has a lower total inorganic mineral content.

### 3.2. Blend Composite Characterization

#### 3.2.1. Mechanical Properties

Table 2 presents the results of the tensile properties of PPLA/Saqqez gum blends with different contents of two plasticizers, ATBC and PEG, in terms of Young’s modulus, elongation at break and yield strength. The amounts were not determined for Saqqez gum, PLA/Saqqez gum (70/30 *w*/*w*) without plasticizer and 14 wt% ATBC samples due to their high brittleness (n.d.). The available data show that the PPLA/Saqqez gum blends with different contents of ATBC and PEG have varying tensile properties. The inclusion of plasticizers seemed to affect the mechanical properties of the blends.

In terms of Young’s modulus, the blends with PLA plasticized with 16% ATBC had the lowest Young’s modulus (1100 ± 0.01 MPa). However, the Young’s modulus was lower in the samples containing 16% and 18% ATBC than in those containing PEG. This suggests that the presence of ATBC improves the stiffness of the blend. Indeed, the addition of plasticizers to a polymer reduces its resistance to deformation, resulting in a lower modulus of elasticity. This is due to an increase in the free volume between the polymer chains, which promotes greater chain mobility and flexibility. Thus, plasticized polymers require less force to deform than polymers without plasticizers [19]. PLA is known for its high fragility and brittle nature, which is characterized by a high Young’s modulus [40]. Therefore, the addition of plasticizers to PLA aims to reduce its fragility [24]. It should be noted that the Young’s modulus of the samples containing 16% and 18% ATBC is lower than that of neat PLA, which has previously been reported to be 3500 MPa [41]. According to Pivsa-Art et al. (2013), the increase in Young’s modulus is due to phase separation that occurs in immiscible compounds [42]. Therefore, it is likely that the ATBC treatments lacked phase separation and demonstrated adequate miscibility.

In terms of elongation at break, the PPLA/Saqqez gum blend with 16% ATBC content has the highest value (130.6 ± 0.1%), followed by the PPLA/Saqqez gum blend with 18% ATBC content (120.4 ± 0.1). Among all samples, the PLA/Saqqez gum with 14% PEG has the lowest elongation at break (80.4 ± 0.1).

By comparison, the elongation of neat PLA, reported to be 8% [43], indicates that all plasticized blends could effectively improve the stretchability of the blend. It is widely recognized that plasticizers lead to increased flexibility and elongation at break of polymers [44,45]. Lim and Hoag (2013) stated that the elongation percentage is a valuable parameter for evaluating the effectiveness of plasticizers based on their type and quantity [19]. Previous studies have shown that the addition of plasticizers, such as PEG and ATBC, can increase the elongation at the break of PLA [23], which is consistent with the results of this study. Additionally, Zhao et al. (2020) found that ATBC had a practical function at a weight increase of 20% [40]. According to Courgneau et al. (2011), the increase in elongation at the break is related to the decrease in Young’s modulus, strength at yield and storage modulus [24].

The results indicate that the PPLA/Saqqez gum blend with 16% ATBC content demonstrates the highest yield strength value (15.4 ± 0.9 MPa), followed by PPLA/Saqqez gum with 14% PEG content (15.2 ± 0.2 MPa). On the other hand, the PLA/Saqqez gum with 16% ATBC exhibits the lowest yield strength value (12.2 ± 0.9). Since then, this value has been reported to be 58 MPa for neat PLA [46], suggesting that the presence of plasticizers, particularly ATBC, reduces the yield strength of the blend compared to neat PLA. Yield strength is defined as the stress required to produce a specific amount of plastic deformation, as stated by Subramaniam et al., (2019) [47]. Kodal et al. (2019) suggest that the inclusion of plasticizers in PLA enhances its ductility and processability. The addition of 10% plasticizer by weight to PLA reduces its Young’s modulus and yield strength while improving its elongation at the break, regardless of the type of plasticizer used. Generally, the yield strength and Young’s modulus of PLA decrease with increasing plasticizer content, unless low molecular weight plasticizer migration to the polymer surface occurs during its service life [43]. Courgneau et al. (2011) reported that PLA and ATBC are miscible up to 17 wt%, but phase separation occurs at concentrations above 5 wt% when blended with PEG [24].

Overall, Table 2 has demonstrated that, in this study, ATBC had a much greater effect on the tensile properties of the blends than PEG, due to the possible phase separation of PEG.

#### 3.2.2. Thermal Properties

**Figure 2:**
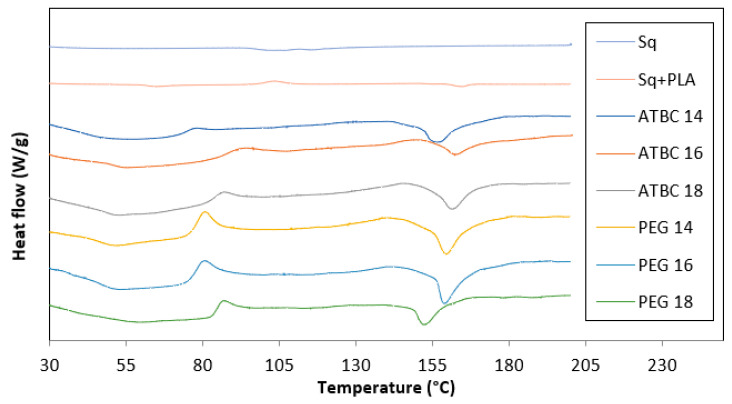
DSC thermograms for Saqqez gum (Sq), PLA/Saqqez gum blend without plasticizer (Sq + PLA) and PPLA/Saqqez gum blends.

The thermal analysis of the prepared treatments was carried out using DSC. The thermal characteristics of the phase changes, including T_g_, T_c_ and T_m_, were recorded and presented in Table 3 and Table 4. The thermogram curves indicated similar thermal behaviors for all samples of PPLA/Saqqez gum blends, as shown in Figure 2. Based on the results, the T_g_ of neat PLA was obtained at 55 °C, which was confirmed by previous studies [48,49]. The T_g_ of Saqqez gum and PLA/Saqqez gum blend without any plasticizer was found to be 58.3 °C and 50 °C, respectively. Table 2 shows that the lowest T_g_ among plasticized blends was 46.2 °C for the blend containing 16% ATBC. The T_g_ of the blend containing 18% PEG was 50.4 °C, similar to the PLA/Saqqez gum blend without plasticizer.

Plasticizers operate by reducing the interactions between the polymer chains, making them more mobile at lower temperatures [19]. Their main thermal effect is to lower the T_g_ by cutting the polymer chain during the process [24]. Plasticizers generally act as modifiers in PLA blends, improving the plasticizing effect and reducing the T_g_, thereby increasing PLA’s ductility [48,50,51,52]. The T_g_ values of pure polymers, polymer blends, copolymers and polymer-based composites reflect their miscibility as a function of composition and determine their properties [53]. Zeng et al. (2015) suggest that the miscibility of a polymer blend can be determined by its morphology (homogeneous or heterogeneous) and the T_g_ of the blend. A homogeneous blend with a single T_g_ between the T_g_ values of both components is considered a completely miscible blend, while a blend with a fine phase morphology and improved properties is referred to as a compatible or partially miscible blend [52]. Therefore, due to the homogeneity of all treatments and a single, lower T_g_ for both components of the blend, they may be considered a partially miscible blend within a range of compatibility.

Fox’s equation describes the glass transition temperature (T_g_) of a miscible compound of a copolymer, two polymers or a plasticized polymer. This equation represents a more accurate free or random volume for the two components in the blends. The predicted T_g_ value in this equation is less than the experimental value given by a simple linear law of compounds [54].

When comparing the T_g_ values obtained from the DSC analysis with the values predicted via Fox’s equation, deviations from the equation were observed. As shown in Table 3, samples without plasticizer and samples containing 14% ATBC showed more deviations, while samples containing 16% ATBC and 18% ATBC, as well as all samples containing PEG, showed less deviations.

According to Lee & Litt (2000) [55], deviations from Fox’s equation in copolymer systems can be explained by steric effects and sequence distributions. However, the effect of polarity on the composition’s T_g_ has not been fully elucidated. Campbell et al. (2000) also believed the systems that do not follow Fox’s equation are less uniform or weak mixtures [54]. In the miscible blends without strong interactions between two polymers, the T_g_ curve usually follows Fox’s equation, whereas in blend systems with strong interactions, such as interchain electron donor–acceptor complex formation or hydrogen bonding, the T_g_ curves show positive deviations from Fox’s equation. Courgneau et al. (2011) investigated the impact of two plasticizers, ATBC and PEG-300, on PLA [24]. The study found that adding up to 17% ATBC by weight reduces the glass transition temperature without causing phase separation, confirming the miscibility of PLA and ATBC. Previous studies have shown that PEG serves as a plasticizer and a compatibilizing agent [56,57], as well as a polymeric surfactant that reduces surface tension and increases interfacial adhesion between dispersed and matrix polymer phases in polymer blends [58]. However, PEG is not as effective as ATBC in reducing T_g_. Courgneau et al. (2011) attributed this function to the phase separation of PEG above 5% [24]. However, some authors have reported phase separation in PEG above 20% [59] and even at 30% by weight [60,61].

According to Qian et al. (2010), the distinct T_g_ of an amorphous mixture is the average T_g_ of its components, indicating homogeneity at the molecular level [62]. However, this approach is limited by the need for a phase separation region larger than approximately 30 nm in DSC measurements of T_g_.

As previously mentioned, plasticizers increase the free space in the polymer network [48]. At certain temperature points, this increased freedom of mobility leads to the spontaneous arrangement of a crystalline structure at the crystallization point. Therefore, the crystallization process is expected to occur earlier and at lower temperatures as the plasticizer content increases. Another effect of adding plasticizers to the polymer is the reduction in the crystallization peak [11]. The results show that all samples have exothermic crystallization points. Increasing the ATBC content from 16 to 18% decreased the crystallization temperature by approximately 7 degrees. Previous studies have confirmed that certain plasticizers, such as PEG and citrate derivatives, can reduce the crystallization temperature [57,63]. According to Abdelwahab et al. (2012), a homogeneous phase is indicated during the heating process when the mixture reaches its crystallization temperature [33]. Additionally, the difference in flexibility of the chains and their ability to form a crystalline structure is related to the change in crystallization peaks.

All samples showed an endothermic peak between 100 and 180 °C in their thermograms. The melting temperature of the treatments changed slightly with increasing amounts of plasticizers. However, compared to the melting temperature of PLA/Saqqez gum without any plasticizer, which was recorded at 155 °C, the treatments with 14% and 16% ATBC showed a decreasing trend.

Wu and Liao (2005) stated that adding plasticizer to the blend is expected to decrease the melting point temperature [64]. However, Abdelwahab et al. (2012) reported no changes in the melting temperature of PLA/PHB (poly hydroxybutyrate) plasticized with Lapol [33]. Previous studies have shown that the endothermic melting temperature is influenced by the molecular motion of the polymer chains and the degradation of the molecular structure. This can be related to the evaporation of moisture from the sample or the degradation of the softener, which can affect the properties of the materials and their application [50,65].

Generally, adding plasticizers (ATBC and PEG) to the blend can effectively alter the crystallization temperature. However, it does not significantly change the glass transition temperature and melting temperature.

#### 3.2.3. Morphological Properties

FESEM was used to analyze the fracture surface morphologies of the samples. This allowed for investigation into the dispersion of Saqqez gum particles, differences between samples with and without plasticizers and the function of plasticizers in the blend. Additionally, the miscibility between the matrices and materials was clarified.

**Figure 3:**
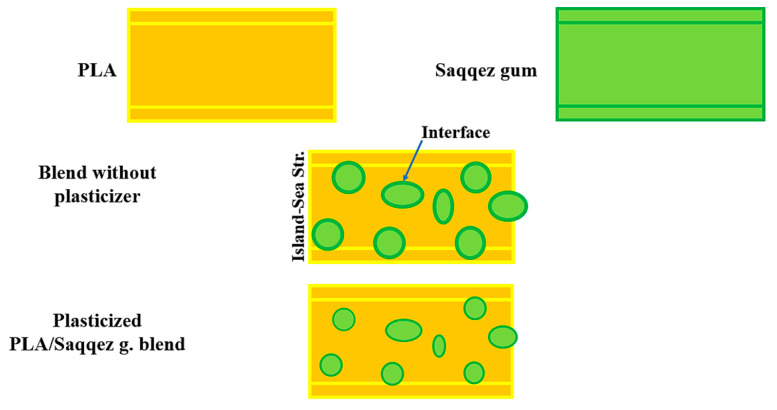
Schematic representations showing the pure PLA, pure Saqqez gum and blends with and without plasticizer showing the effect of plasticization in the blend structure.

**Figure 4:**
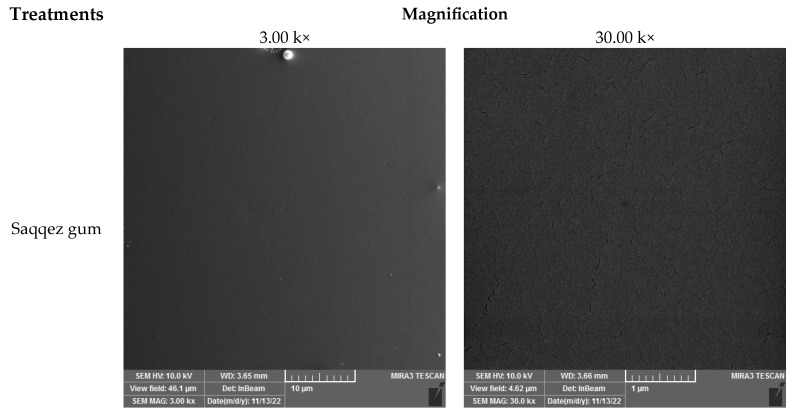

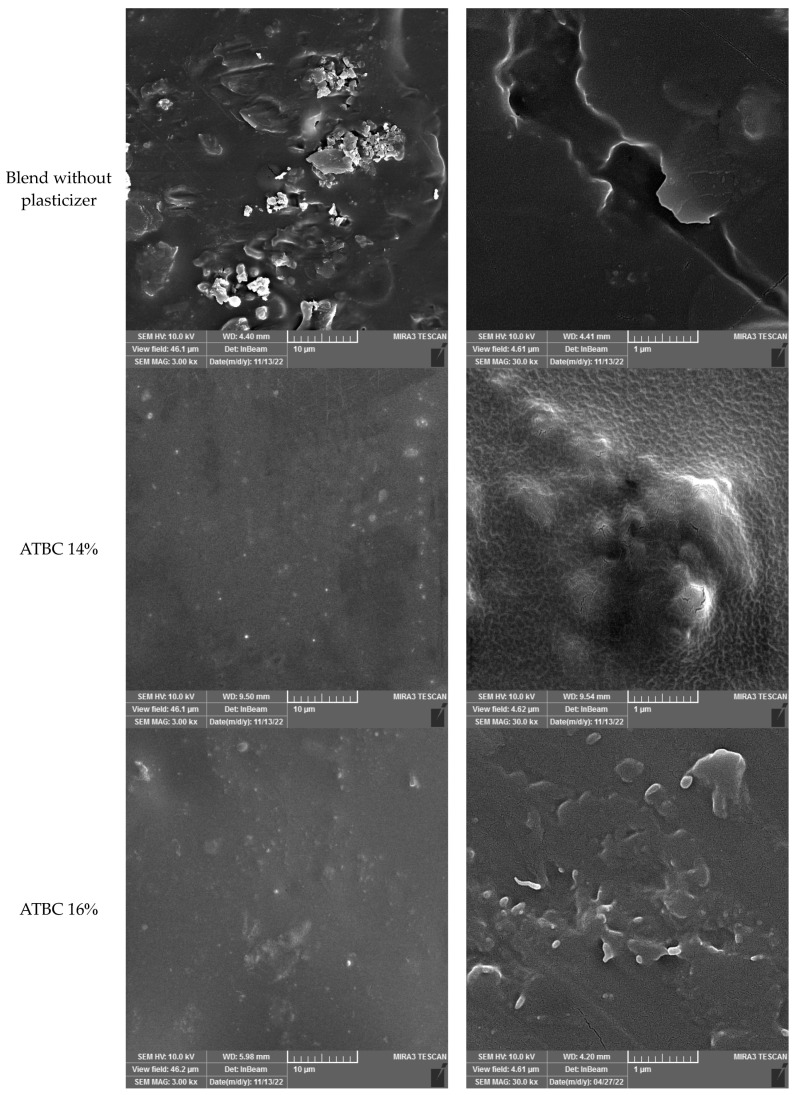

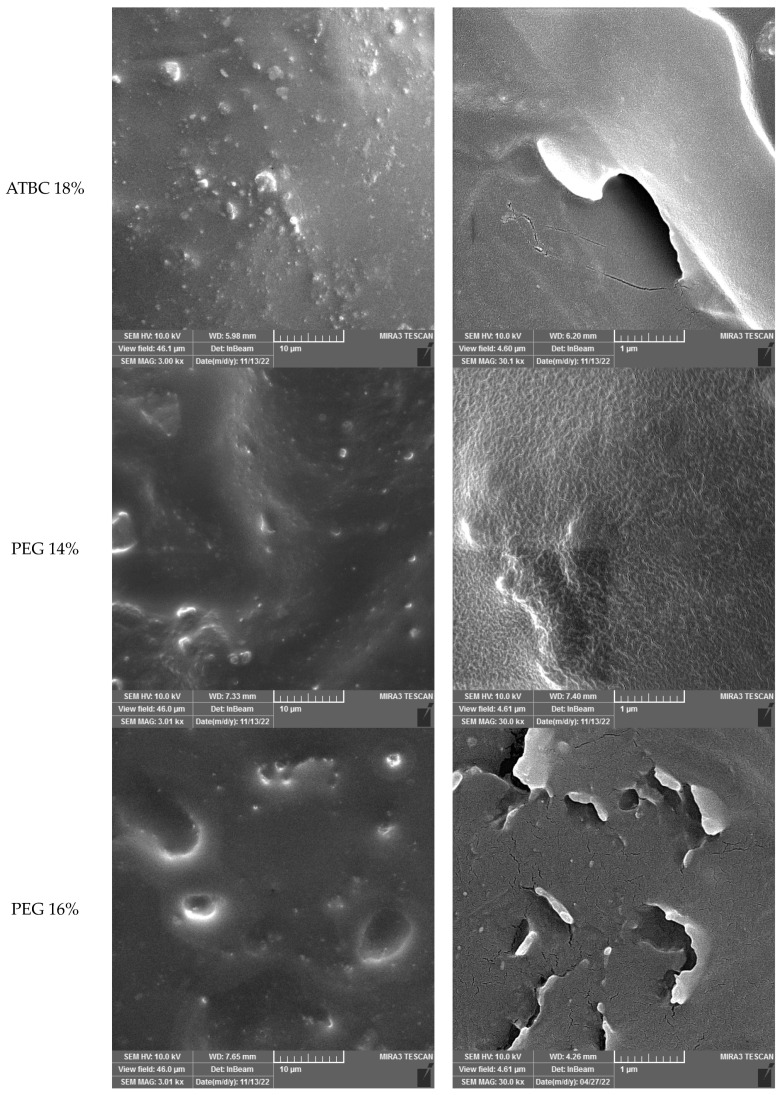

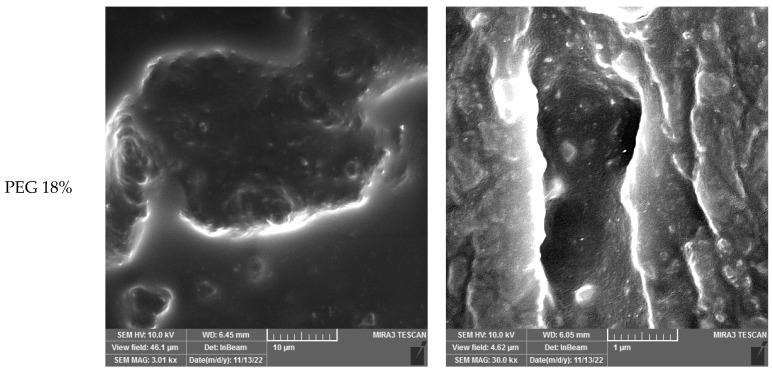
SEM micrographs of the Saqqez gum, blend without plasticizer and plasticized blends containing ATBC and PEG in different contents at the indicated magnification.

According to Mao et al. (2019), the properties of polymer blends, including toughness, rigidity and thermal resistance, are not solely determined by the properties of each component, but also by the morphology, specifically the shape and distribution of the components [66]. To better understand the structure of the blend and evaluate the micrographs, it is important to state the role of each component and the effect of plasticization. Figure 3 provides visual illustrations of this. Saqqez gum particles are used to toughen the brittle polymeric matrix of PLA, act as stress concentrators in the blend and induce a large number of crazes during the deformation process. Polymer blends can sometimes be structured as sea-island or core-shell structures, where two phases are present as discrete spherical domains embedded in a surrounding matrix. In this study, Saqqez gum was dispersed as the “island” or minor phase in PPLA and PPLA as the “sea” phase in blends. This structure was confirmed via SEM micrographs presented in Figure 4. Previous studies have shown that the fracture surfaces of neat PLAs are smooth [67], and form the major continuous phase [67,68,69]. SEM images also indicate that the Saqqez gum surface is smooth and cavity-free. Wei et al. (2021) found that immiscible polymer blends exhibit various morphologies, including sea-island structures, depending on the ratio of polymer compounds, interfacial adhesion and processing conditions [70]. The addition of a plasticizer to the PLA matrix is suggested to improve interfacial adhesion and dispersion. Chemical interactions between the components resulted in a diffused interface and good adhesion, leading to a homogeneous matrix without separation at the interface. This indicates plasticization in the PLA matrix [71]. In addition, plasticizers can significantly reduce the size of island agglomerates and increase the dispersion between the blend components [72], as shown in Figure 3. It is widely acknowledged that smaller particle sizes facilitate improved interfacial interactions, resulting in better mechanical properties of blends [69], as confirmed by the results of tensile tests conducted on plasticized samples.

Micrographs of the sample without plasticizer reveal significant heterogeneity in the blend, with fractures and cavities displaying sharp edges that indicate the presence of sea-island structures. The Saqqez gum domains, which have a broad size and distribution and an irregular shape, are dispersed throughout the PLA matrix. By adding the plasticizers, the homogeneity of the samples containing both plasticizers was significantly increased compared to the sample without plasticizer.

A good homogeneity is observed in the samples containing ATBC (14 and 16%). This finding is consistent with the results reported by Yu et al. (2008) in their study of PLA/ATBC/CB (carbon black), where ATBC was found to reduce the size of CB agglomerates [29]. However, it appears that the proportion of sea-island structures increased by 18%, and they also grew in size, resulting in more distinct interfaces between the continuous phase and dispersed Saqqez particles. Previous studies have confirmed that the addition of ATBC leads to an increase in the diameter of the dispersed phase [73,74]. According to Aliotta et al. (2021), the addition of plasticizer reduces viscosity, which in turn increases the diameter of the dispersed domain [73]. In blends with 15 wt% or more of ATBC content, the dispersed particles become closer to each other, forming larger domains. In samples containing PEG, similar to ATBC, Saqqez domains (dispersed phase) were homogeneously dispersed in a smaller size PLA matrix in 14% and 16% of samples compared to the sample without plasticizer. In a sample containing 18% PEG, heterogeneity occurred. Previous studies have suggested that this may be related to phase separation in mixtures containing PEG above 17% [24]. The micrographs revealed that the Saqqez gum droplets coalesced during melt-blending, even at 18 wt% of ATBC and PEG. This suggests a range of miscibility.

#### 3.2.4. Water-Absorption Behavior

**Figure 5:**
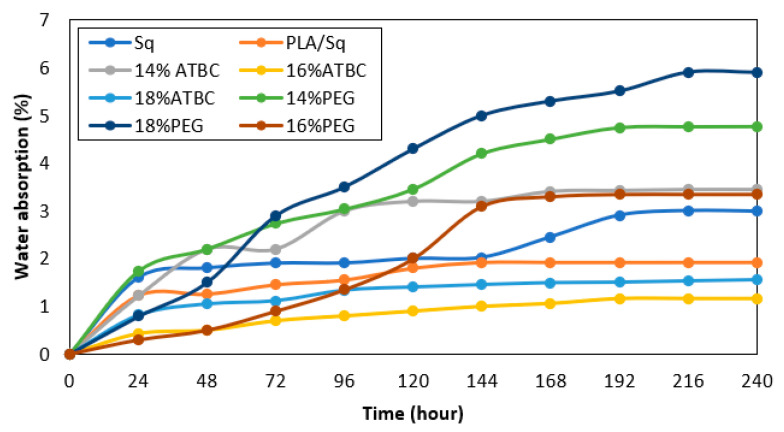
Water absorption (%) of Saqqez and treated samples versus time.

Table 5 presents data on the coefficients of water absorption, sorption, diffusion and permeability coefficients of the samples after 240 h (10 days). The highest and lowest values of water absorption in the saturation stage (%) were associated with PEG 18% and ATBC 16%, respectively. It can be inferred that ATBC 16% exhibits the greatest resistance to environmental moisture. This absorption behavior is also evident in all samples, as illustrated in Figure 5. However, with the addition of plasticizer, the water-absorption gradually increased, except for 16%, which could be due to the greater cohesion of its structure compared to the other treatments. The water absorption of 18% may indicate an incoherent structure, which is supported by the results of other tests in this investigation. However, it could also be a sign of a higher water-absorption capacity in this sample. The measurement of water absorption in materials determines the quantity of water they absorb, while water sorption refers to their capacity to attract and retain water molecules. Both parameters are related to the interaction between water and the material. Water sorption affects the water-absorption capacity of a material. The sorption coefficient (S) represents the ability of a substance to sorb or adsorb water. Therefore, higher values indicate a greater sorption capacity. The study results show that PEG 18% has the highest value, while ATBC 16% has the lowest value, similar to water absorption.

Similarly, the highest and lowest values for the diffusion coefficient (D) and the permeability coefficient (P) belong to PEG 18% and ATBC 16%, respectively. These coefficients represent the rate at which water molecules penetrate through the samples and the ease of water flow through the samples, respectively.

The study confirms that a sample with a higher water-absorption capacity is likely to have higher sorption, diffusion and permeability coefficients.

A thorough review of the water-absorption properties of natural fiber-reinforced PLA composites was conducted by Rahman & Mustapa (2021) [75]. In addition, Kamaludin et al. (2021) investigated the water-absorption kinetics of PLA/chitosan composites and found that the addition of chitosan increased water uptake, suggesting a greater interaction between water and filler, which in turn led to a decrease in tensile properties [76]). In another investigation on the water-absorption behaviour of cellulose-reinforced PLA biocomposites, Penjumras et al. (2015) reported that water absorption increased with increasing cellulose content and exposure time [77].

#### 3.2.5. Chemical Resistance

Table 6 presents data on the chemical resistance of Saqqez and polymer blends following exposure to 1 mol/L HCl and 1 mol/L NaOH for 240 h, along with the corresponding weight percentages (+/−) for each blend. The results for HCl indicate weight gain in all compounds except for Saqqez, which experienced weight loss. It can be concluded that blending Saqqez and PLA increases resistance in Saqqez. Regarding the blends, they not only did not lose weight but also increased in swelling. The weight increase indicates that the blends experienced swelling due to the inclusion of HCl, rather than erosion. In general, no weight loss was observed regarding the resistance to NaOH. It is noteworthy that in the plasticizer blends, the increase gradually reduces with the increase in the content of plasticizers. Guduri et al. (2007) observed that the weight gain percentage was higher in Hildegardia fabric/polycarbonate-toughened epoxy composites treated with NaOH. This is believed to be due to the increased exposure of OH groups in cellulose, leading to greater hydrophilicity of the system [78]. Wahit et al. (2015) discovered that the chemical resistance of PLA decreased with an increase in the content of Epoxidized Natural Rubber [79].

### 3.3. Comparative Chewing Gum Tests

#### 3.3.1. Sensory Evaluation

According to the results presented in Table 7, the overall acceptance is equal in both samples. However, there is a slight difference between the two samples in terms of texture and appearance, with the produced sample scoring higher in color and smell.

#### 3.3.2. Texture Profile Analysis (TPA) Test

As can be seen from the results (Table 8), the hardness of the desired sample is higher than that of the commercial sample, which also affected the chewability, which was predictable given the use of natural gum instead of commercial elastomer in the manufacture of chewing gum. This is because the degree of cohesion between the two samples is not very different. On the other hand, the springiness of the desired sample is greater than that of the commercial sample, which can have a major impact on overall consumer acceptance. Similarly, the rupture point, which indicates the ease of fragility of the chewing gum during chewing, should ideally have a lower numerical value; the desired chewing gum sample has a lower value than the commercial sample.

## 4. Conclusions

In conclusion, the mechanical properties of PPLA/Saqqez gum blends with plasticizers (ATBC and PEG) have shown significant variations. The addition of plasticizers impacted the tensile properties, with ATBC demonstrating a more pronounced effect than PEG. Blends with ATBC showed improved stiffness, elongation at break and yield strength, while PEG contributed to enhanced stretchability. The thermal properties were also influenced, with plasticizers reducing the glass transition temperature. Morphological analysis revealed changes in homogeneity and size of dispersed phases. Additionally, water absorption and chemical resistance were affected by plasticizer content, with ATBC 16% demonstrating greater resistance to moisture and chemicals. Comparative chewing gum tests revealed differences in texture and hardness, with the desired sample exhibiting higher springiness and lower fragility during chewing. In terms of successful applications and the development of similar chewable gum products using this biocomposite, future research could focus on enhancing its flavor release, texture and shelf life to match or exceed traditional chewing gum. Additionally, investigating its potential in oral care products, controlled-release nutraceutical gums and functional chewing gums tailored for specific health benefits could be promising areas for future research and development. Considering the high potential of the blends studied in this survey in terms of flexibility and thermal properties, future research will focus on the biodegradability and application of this emerging and promising compound in the other food and packaging industries as a thermoplastic elastomer.

## Figures and Tables

**Table 1:** The chemical composition values of Saqqez gum.

Chemical Composition	Content (%)
Total Ash	0.84 ± 0.14
Moisture	16.3 ± 0.02
Fat	1.80 ± 0.23
Protein	9.14 ± 0.01
Polysaccharide	86.89 ± 0.11

**Table 2:** Tensile properties of Saqqez gum, PLA/Saqqez gum (70/30 *w*/*w*) without plasticizer and PPLA/Saqqez gum with different contents of ATBC and PEG.

	Plasticizer Content (wt%)	Young’s Modulus (MPa)	Elongation at Break (%)	Yield Strength (MPa)
Saqqez	-	n.d. *	n.d. *	n.d. *
PLA/Saqqez **	-	2650 ± 22.3 ^a^	7.7 ± 0.9 ^f^	32.4 ± 1.3 ^a^
PPLA/Saqqez (ATBC)	14	1430 ± 26.1 ^bc^	75.6 ± 3.3 ^e^	18.3 ± 0.8 ^b^
	16	1100 ± 70.3 ^c^	130.6 ± 6.1 ^a^	12.2 ± 0.9 ^e^
	18	1200 ± 62.6 ^c^	120.4 ± 1.0 ^b^	13.3 ± 0.4 ^f^
PPLA/Saqqez (PEG)	14	2300 ± 72.1 ^a^	80.4 ± 2.3 ^d^	15.2 ± 0.2 ^c^
	16	2200 ± 36.5 ^a^	94.6 ± 6.3 ^c^	15.4 ± 0.9 ^c^
	18	1500 ± 28.9 ^b^	90.1 ± 1.2 ^c^	14.2 ± 0.4 ^d^

Means labeled with the same letter are not significantly different (*p* < 0.05). * Not determined. ** Without plasticizer.

**Table 3:** Experimental T_g_ and T_g_ predicted via Fox’s equation for PPLA/Saqqez gum blends with different contents of ATBC and PEG.

Samples	Experimental T_g_ (°C)	Predicted T_g_ (°C)
Saqqez gum	58.3	-
PLA	55.0	-
Blend without plasticizer	50.0	59.0
ATBC 14%	48.2	49.6
ATBC 16%	46.2	37.5
ATBC 18%	47.9	42.4
PEG 14%	48.7	41.4
PEG 16%	47.1	35.5
PEG 18%	50.4	40.4

**Table 4:** Thermal parameters of Saqqez gum, PLA, PLA/Saqqez blend without plasticizer and PPLA/Saqqez gum blend plasticized with different contents of ATBC and PEG.

Samples		T_c_ (°C)	T_m_ (°C)
**Saqqez gum**		90.9	102.9
**PLA**		120.0	160.0
**Blend without plasticizer**		109.0	155.7
**Plasticized blends**	ATBC 14%	78.4	141.5
	ATBC 16%	94.4	146.8
	ATBC 18%	87.4	161.5
	PEG 14%	80.8	159.6
	PEG 16%	80.9	159.1
	PEG 18%	87.2	152.4

**Table 5:** Water absorption, sorption, diffusion and permeability coefficient of samples at 240 h.

Sample	Water Uptake at Saturation Stage (%)	Diffusion Coefficient (D) (mm^2^/s)	Sorption Coefficient (S)	Permeability Coefficient (P) (mm^2^/s)
Saqqez gum	2.902	0.984	0.821	0.808
Blend without plasticizer	2.760	0.722	0.806	0.582
ATBC 14%	3.448	1.128	0.928	1.046
ATBC 16%	0.830	0.408	0.774	0.315
ATBC 18%	3.466	0.520	0.860	0.447
PEG 14%	3.668	1.658	0.943	1.564
PEG 16%	2.608	1.139	0.925	1.053
PEG 18%	6.020	1.997	1.005	1.998

**Table 6:** Chemical resistances (in terms of %wt. loss/gain) of Saqqez and polymer blends at 240 h against 1 mol/L HCl and 1 mol/L NaOH.

Pcr (%)	Sq	PLA/Sq	14%ATBC	16%ATBC	18%ATBC	14%PEG	16%PEG	18%PEG
HCl	−0.880	+1.604	+2.863	+2.216	+1.699	+5.803	+5.040	+5.003
NaOH	+6.983	+1.536	+8.570	+2.115	+0.079	+12.440	+13.850	+0.907

Weight gain (+)/loss (−).

**Table 7:** Comparing sensory evaluation of the chewing gum sample with the desired elastomer and commercial chewing gum.

Sample	Appearance	Texture	Odor	Color	Overall Acceptance
Produced chewing gum	3.6 ^b^	3.3 ^b^	4.6 ^a^	3.3 ^a^	4.0 ^a^
Commercial chewing gum	4.0 ^a^	4.0 ^a^	3.0 ^b^	3.0 ^a^	4.0 ^a^

Means labeled with the same letter are not significantly different (*p* < 0.05).

**Table 8:** TPA test results of the chewing gum sample with the desired elastomer and commercial chewing gum.

Sample	Hardness	Cohesiveness	Springiness	Chewiness	Rupture Point
Produced chewing gum	3165.18 ^a^	0.3723 ^b^	1.0041 ^a^	1240.261 ^a^	6528.542 ^b^
Commercial chewing gum	1941.13 ^b^	0.4325 ^a^	0.4024 ^b^	162.211 ^b^	8525.270 ^a^

Means labeled with the same letter are not significantly different (*p* < 0.05).

## Data Availability

The data presented in this study are available on request from the corresponding author.
